# A universal mammalian vaccine cell line substrate

**DOI:** 10.1371/journal.pone.0188333

**Published:** 2017-11-27

**Authors:** Jackelyn Murray, Kyle V. Todd, Abhijeet Bakre, Nichole Orr-Burks, Les Jones, Weilin Wu, Ralph A. Tripp

**Affiliations:** Department of Infectious Diseases, College of Veterinary Medicine, University of Georgia, Athens, GA, United States of America; University of Hong Kong, HONG KONG

## Abstract

Using genome-wide small interfering RNA (siRNA) screens for poliovirus, influenza A virus and rotavirus, we validated the top 6 gene hits PV, RV or IAV to search for host genes that when knocked-down (KD) enhanced virus permissiveness and replication over wild type Vero cells or HEp-2 cells. The enhanced virus replication was tested for 12 viruses and ranged from 2-fold to >1000-fold. There were variations in virus-specific replication (strain differences) across the cell lines examined. Some host genes (CNTD2, COQ9, GCGR, NDUFA9, NEU2, PYCR1, SEC16G, SVOPL, ZFYVE9, and ZNF205) showed that KD resulted in enhanced virus replication. These findings advance platform-enabling vaccine technology, the creation of diagnostic cells substrates, and are informative about the host mechanisms that affect virus replication in mammalian cells.

## Introduction

Vero cells, established from an African green monkey (non-human primate; NHP) are commonly used during poliovirus (PV), rotavirus (RV) and in some influenza A virus (IAV) vaccines [1–10]. A fortuitous deletion corresponding to the type 1 interferon (IFN) loci renders Vero cells susceptible to many viruses [11]. Egg-based vaccine production is the current method for vaccines like IAV, but is time-consuming, expensive and cannot respond rapidly to newly emerging strains. Mammalian cell culture-based vaccines can overcome these limitations, allowing for rapid scale up of vaccines, reduce production and purification costs, and exhibit equivalent or higher vaccine and efficacy in both immunocompetent and allergic individuals. As such, Vero cells are being used to make seasonal IAV vaccines due to variants that arise by antigenic drift [12]. One option for developing influenza vaccines is to create a vaccine cell line that is permissive for a large cohort of influenza subtypes [12]. Infection with a range of IAVs will promote reassortment of viral segments, leading to a mixture of diverse species, which can be used for vaccination. Achieving this capability could transform vaccinology.

Viruses co-opt host cells for their replication. Viruses must employ aspects of the host cellular machinery to translate viral mRNA, and to avoid host defenses in order to replicate. Additionally, viral strategies to replicate include the use of non-coding RNAs, the manipulation of translation factors, as well as modification of host cell microRNAs that regulate genes [13, 14]. Using small interfering RNA technology (siRNA) it is possible to knock-down (KD) host genes affecting virus replication allowing one to determine pro- and anti-viral genes [15]. A general approach in creating a universal vaccine cell line is to ablate host cell defenses, and/or create or improved cell tropism. Thus, the host-resistance genes to poliovirus (PV), influenza A virus (IAV) and rotavirus (RV) in Vero cells were identified using siRNAs [1, 3, 16–18]. The results show that one can increase virus replication without altering antigenicity [1, 18, 19]. Once the host genes needed for virus replication are validated, it is possible with clustered regularly interspaced short palindromic repeat (CRISPR)-Cas9 reagents to knock-out (KO) host genes with goal of gene editing cells to improve viral antigen and/or increase viral replication with the long-term goal of creating enhanced vaccine cells lines [1, 3].

The studies examined here determined if KD of PV genes [1], IAV genes [17, 18], or RV genes [3] (Tables 1–2) would increase virus replication as determined by plaque assay in either Vero cell or HEp-2 cells (Table 3). An aim was to determine if there were host genes which when silenced would enhance virus replication. We tested several RNA viruses including influenza A/WSN/1933 (IAV), poliovirus Sabin-2 (PV), rotavirus/G3P[6]/BB (RV), influenza B/Malaysia/2506/2004 (IBV), Dengue virus type 1, Hawaii (DENV), Yellow fever virus 17 D vaccine (YFV), Hepatitis A virus (HAV), Coxsackie virus B5 (EV71), mumps virus (MuV), rubella virus (RUBV), and a DNA virus, Varicella zoster virus (VZV). The results identified genes that when KD allowed for enhanced viral permissiveness and replication as determined by plaque assay or ELISA across viral families. Specifically, there were several host genes which enhanced virus replication when CNTD2, COQ9, GCGR, NDUFA9, NEU2, PYCR1, SEC16G, SVOPL, ZFYVE9, and ZNF205 genes were KD in Vero cells (Table 3). The findings suggest that KD of many of the genes examined (Table 1) might be useful to make a universal cell line, though KD of more than one gene identified was not examined in this study.

**Table 1:** Top 6 genes that alter replication of poliovirus (PV), rotavirus (RV) or influenza A virus (IAV).

Virus	Gene name	Gene symbol
PV	butyrophilin subfamily 2 member A1	BTN2A1
	cyclin N-terminal domain containing 2	CNTD2
	E1A binding	EP300
	glucagon receptor	GCGR
	sec61 translocon gamma subunit	SEC61G
	zinc finger protein 205	ZNF205
RV	coenzyme Q9	COQ9
	N-acetyl transfer 9 (putative)	NAT9
	NADH: Ubiquinone oxidoreductase subunit A9	NDUFA9
	neuraminidase 2	NEU2
	synaptic vesicle-2 related protein like	SVOPL
	RAD51 associated protein	RAD51AP1
IAV	coenzyme Q9	COQ9
	NADH: Ubiquinone oxidoreductase subunit A9	NDUFA9
	phospholipase A2 group 1B	PLA2G1B
	pyrroline-5-carboxylate reductase 1	PYCR1
	zinc finger FYVE- type containing 9	ZFYVE9
	zinc finger protein 205	ZNF205

The top six genes that were identified from a genome-wide siRNA screen for poliovirus (PV), rotavirus (RV) and influenza A virus (IAV).

**Table 2:** qRT-PCR validation of siRNA-mediated gene KD in Vero cells.

	Percent expression				
	NTC		siRNA treatment		Adjusted P Value
	Replicate 1	Replicate 2	Replicate 1	Replicate 2	
BTN2A1	100.00	100.00	0.03	0.03	<0.0001
CNTD2	100.00	100.00	0.00	0.04	<0.0001
COQ9	100.00	100.00	0.00	2.37	<0.0001
EMX2	100.00	100.00	0.00	1.57	<0.0001
EP300	100.00	100.00	0.00	0.86	<0.0001
FGF2	100.00	100.00	0.00	3.67	<0.0001
GCGR	100.00	100.00	0.11	0.05	<0.0001
NAT9	100.00	100.00	0.00	1.08	<0.0001
NDUFA9	100.00	100.00	0.00	0.88	<0.0001
NEU2	100.00	100.00	0.00	0.00	<0.0001
PLA2G1B	100.00	100.00	0.00	0.00	<0.0001
PYCR1	100.00	100.00	0.00	0.01	<0.0001
RAD51AP1	100.00	100.00	0.00	0.00	<0.0001
SEC61G	100.00	100.00	0.00	0.02	<0.0001
STRADA	100.00	100.00	0.00	0.00	<0.0001
SVOPL	100.00	100.00	0.00	0.00	<0.0001
ZFYVE9	100.00	100.00	0.00	0.00	<0.0001
ZNF205	100.00	100.00	0.00	0.02	<0.0001

Vero cells were transfected with siRNAs against listed genes (50nM). Total RNA was isolated and treated with DNAse I to remove genomic DNA, reverse transcribed using oligo dT, and expression determined relative to GAPDH using custom Vero-specific primers for each gene. Amplification conditions were optimized as needed and amplicons were validated via Sanger sequencing. BTN2A1—butyrophilin subfamily 2 member A1; CNTD2—cyclin N-terminal domain containing 2; COQ9—coenzyme Q9; EMX2—empty spiracles homeobox 2; EP300—E1A binding protein; FGF2—fibroblast growth factor 2; GCGR- glucagon receptor; NAT9- N-acetyl transferase 9 (putative); NEU2-Neuraminidase 2; PLA2G1B - phospholipase A2 group IB; PYCR1—pyrroline-5-carboxylate reductase 1; RAD51AP1—RAD51 associated protein 1; SEC61G - SEC61 translocon gamma subunit; SVOPL—SVOP like protein; ZFYVE9—zinc finger FYVE-type containing 9; ZNF205—Zinc finger protein 205. Gene expression is shown as percent. All comparisons were done using 2-way ANOVA and post hoc-Sidak test at α = 0.05.

**Table 3:** siRNA gene KD modulates replication of several viruses.

	Vero cells	HEp-2 cells
*BTN2A1*	PV, DENV, MuV, ZIKV*	MuV, YFV
*CNTD2*	PV, HAV	EV71, HAV
*COQ9*	YFV, HAV	DENV, IBV*
*EMX2*	RV	EV71, HAV
*EP300*	RC, EV71, YFV, VSV	EV71, HAV, IBV*
*GCGR*	PV, YFV, HAV, ZIKV*	
*PLA2GB1*	YFV	EV71
*PYCR1*	IAV, HAV, ZIKV*	HAV, VZV
*NAT9*	RV, ZIKV*	UBV*
*NDUFA9*	RV	HAV, IBV*
*NEU2*	HAV	
*RAD51AP1*	YFV, ZIKV*	
*SEC61G*		HAV
*STRADA*	DENV	
*SVOPL*	DENV, MuV	
*ZNF205*	RUBV, ZIKV*	HAV
*ZFYVE9*	DENV, ZIKV*	

BTN2A1- butyrophilin subfamily 2 member A1; CNTD2- cyclin N-terminal domain containing 2; COQ9—ubiquinone biosynthesis protein, EMX2—homeobox-containing transcription factor, EP300- E1A binding protein; GCGR—G-protein coupled receptor for glucagon, PLA2GB1- epithelial-cell-derived group 1B phospholipase A2, PYCR1- pyrroline-5-carboxylate reductase 1; NAT9—N-Acetyltransferase 9, NDUFA9—NADH-coenzyme Q Reductase, NEU2—neuraminidase 2, RAD51AP1—RAD51 associated protein, SEC61G - SEC61 translocon gamma subunit; STRADA—STE20-related kinase adapter protein alpha, SOPL—a paralog of the SVOP gene that encodes synaptic vesicle 2-related protein; ZNF205—zinc finger protein 205; ZFYVE9—zinc finger FYVE domain-containing protein 9, IAV-Influenza A virus; PV—poliovirus; RV- rotavirus 3; IBV—influenza B virus; DENV—dengue virus type 1; YFV—Yellow fever virus 17D strain; HAV—hepatitis A virus; EV71—Coxsackie virus B5; MuV -mumps virus, RUBV—rubella virus; VZV—varicella zoster virus. Based on significantly different error bars representing ±SEM from three independent experiments p<0.001.

* = results from one independent plaque assay where n = 3 wells/gene KD were evaluated and the fold-change over the NTC.

## Results

The ability to connect systems biology with host gene discovery to aid our understanding of the virus-host network is essential for creating a universal vaccine cell line. We used complementary approaches and data sets from several high-throughput screens for IAV [18], PV [1] and RV [1, 3] to arrive at a multidimensional view of virus-host networks. We screened the 6 top host genes for PV, RV or IAV that enhanced virus replication (Table 1), and tested if gene KD using siRNAs in Vero cells or HEp-2 cell lines would affect host cell permissiveness and allow for enhanced virus replication for 12 different viruses compared to control WT cells or cells transfected with a non-targeting (NTC) control siRNA. All siRNAs tested were ON-TARGETplus siRNAs designed to be seed-region optimized, leading to minimal off-target effects [20]. The genome-wide screens for PV, IAV and RV showed no or negligible off-target effects. All host genes targeted for KD in Vero cells or HEp-2 cells were ≥95% silenced before virus infection (Table 2).

**Fig 1:**
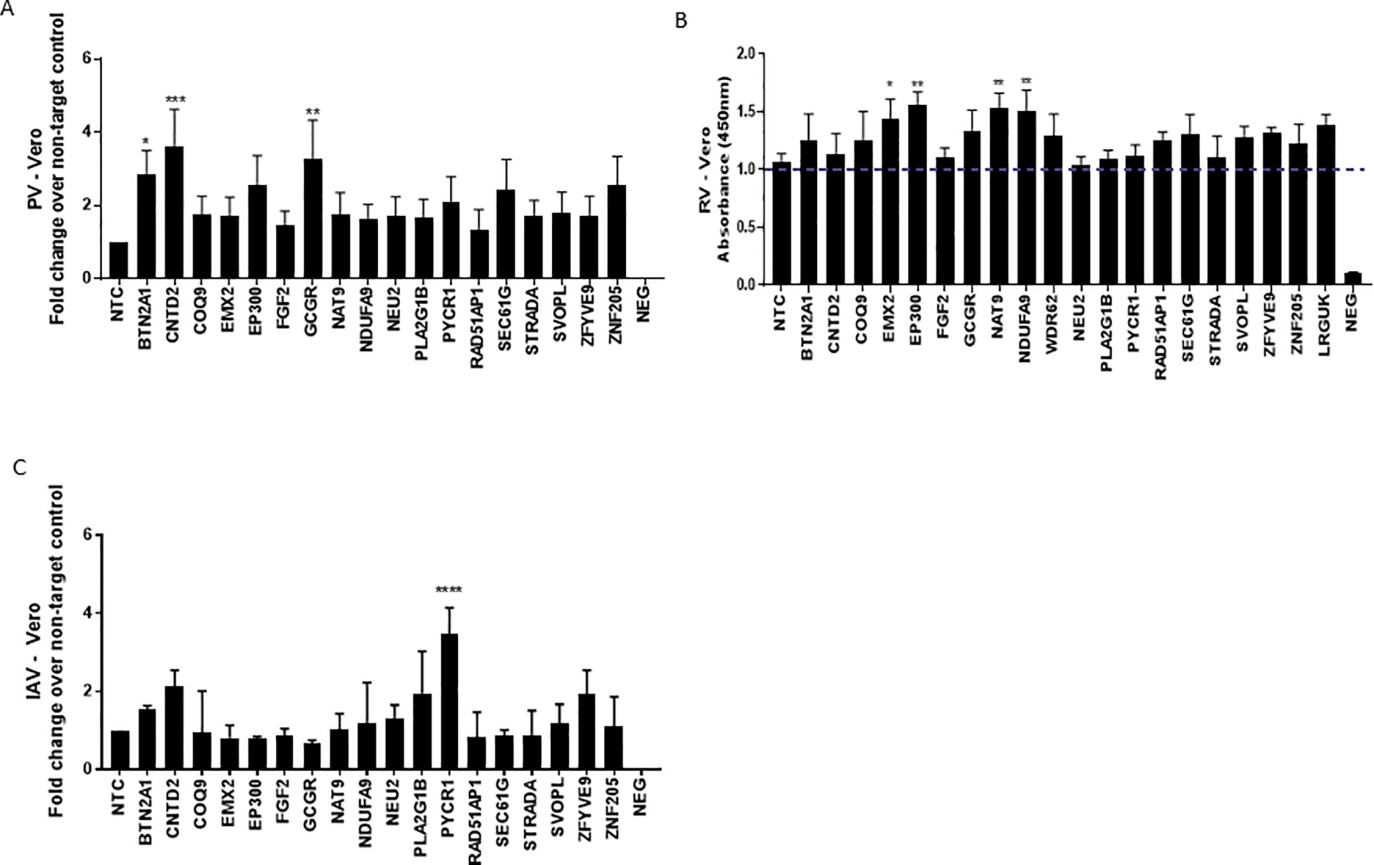
Genome-wide siRNA screening identifies 18 genes that modulate replication of poliovirus (PV), rotavirus (RV) or influenza A virus (IAV). Vero cells were reverse transfected with non-targeting control (NTC) / gene specific on-target plus (OTP) siRNAs (50nM) for 48h followed by infection with PV (A), RV (B) or IAV (C). Effect of gene knockdown on viral replication is shown as fold-change relative to NTC from pooled samples. Data represent plaque numbers (A and C) and absorbance (λ = 450nm) for (B). Error bars represent ±SEM from three independent experiments. * p<0.05 **p<0.01 ***p<0.001 ****p<0.0001.

For these studies, the PV strain was Sabin-2 as the World Health Organization recently endorsed elimination of Sabin -1 strains [2]. We noted virus strain differences in the magnitude of PV replication while switching from Sabin-1 to Sabin-2, particularly in the use of host genes involved in the ability of these strains to replicate which we do not fully understand [21]. In Vero cells, the highest PV replication resulted from KD of CNTD2 (~4-fold) followed by KD of BTN2A1 and GCGR (~3-fold), and EP300, NAT9, RAD51AP1, SEC61G and ZNF205 that ranged from ~2- increases (Fig 1A). In HEp-2 cells, the highest PV replication occurred for KD of CNTD2 (3-fold), where BTN2A1, EP300, GCGR, and ZNF205 KD resulted in >2-3-fold increases. We also examined the ELISA results for RV (Fig 1B). An ELISA was necessary since RV does not plaque well in Vero cells [22, 23]. The highest absorbance (480 nm) was for KD of EMX2, EP300, NAT9 and NDUFA9 genes which was significantly above NTC background. We also evaluated the top 6 genes for IAV replication in Vero cells (Fig 1C). IAVs are divided into subtypes based on two proteins on the surface of the virus: the hemagglutinin (H) and the neuraminidase (N). There are 18 different hemagglutinin subtypes and 11 different neuraminidase subtypes [24]. The top genes identified that when KD increased IAV replication were CNTD2, PLA2G1B and PYCR1 (2-4-fold) followed by ZFYVE9 (~2-fold).

We examined five RNA virus families (*Picornaviridae*, *Flaviviridae*, *Togaviridae*, *Orthomyxoviridae*, *Reoviridae*, *and Paramyxoviridae)* and 1 DNA virus family, *i*.*e*. *Herpesviridae* in our efforts to develop a universal cell line. All of these viruses were tested in Vero cell and HEp-2 cell lines that were targeted by siRNA to KD the top 6 gene hits (Table 3).

**Fig 2:**
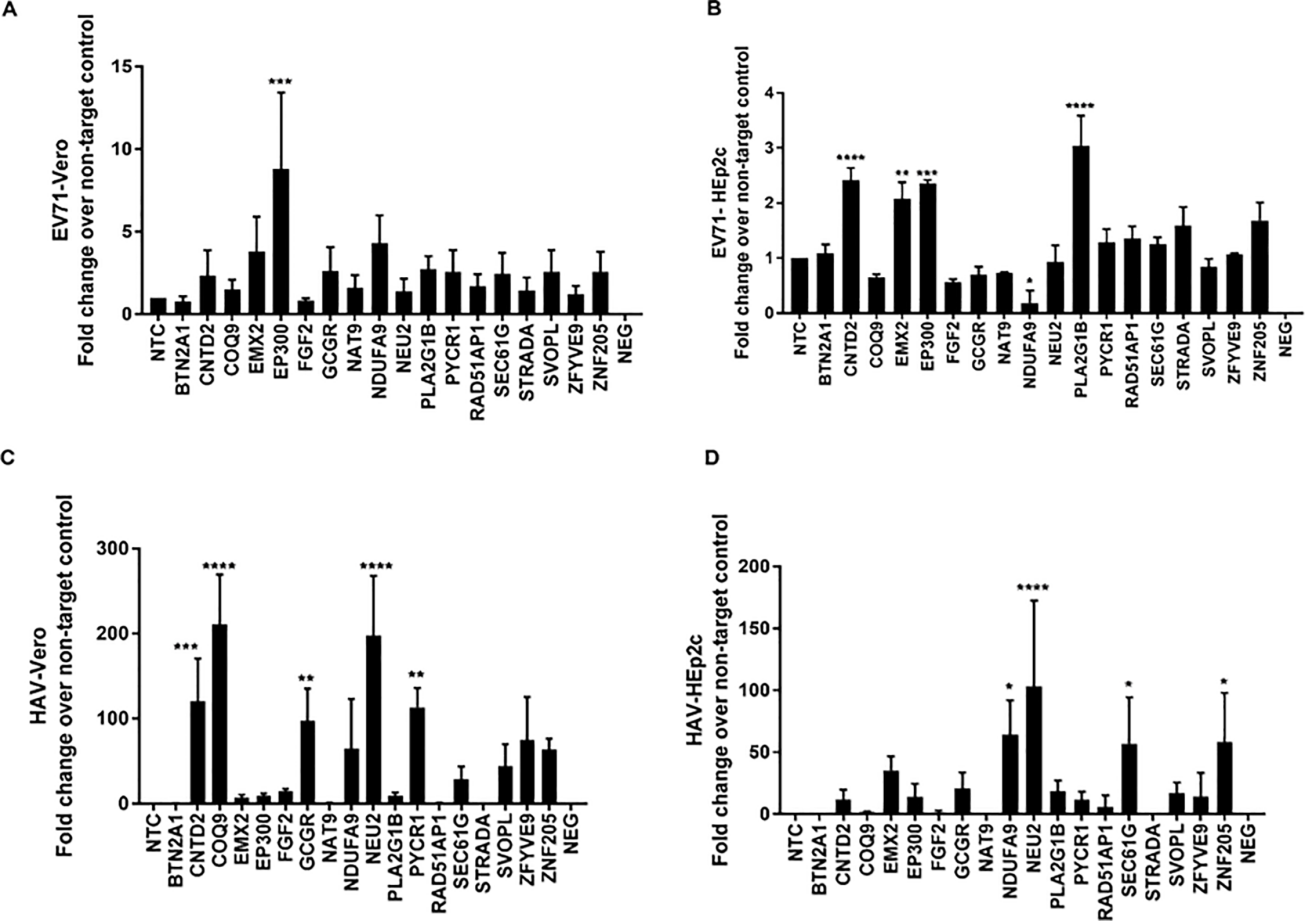
siRNA identifies cell type specificity of genes that modulate replication of EV71 and HAV. Vero / HEp2-c cells were reverse transfected with non-targeting control (NTC) / gene specific on-target plus (OTP) siRNAs (50nM) for48h followed by infection with EV71 (A and B) or Hepatitis A virus (HAV) (C and D). Effect of gene knockdown on viral replication is shown as fold -change relative to NTC from pooled samples. Error bars represent ±SEM from two independent experiments. * p<0.05 **p<0.01 ***p<0.001 ****p<0.0001.

EV71 is a picornavirus [25] and was propagated in Vero cells (Fig 2A) and in HEp-2 cells (Fig 2B). In Vero cells, there was a ~ 8-fold increase in virus replication following KD of host gene EP300. As the results are expressed as fold-change over NTC, all ≥2-fold increase in replication was considered notable. Other notable host genes affecting EV71 replication were CNTD2, EMX2, GCGR, NAT9, NDUFA9, PLA2G1B, PYCR1, SEC61G, SOPL and ZNF205 (Fig 2A). For HEp-2 cells, KD of CNTD2, EMX2, EP300, and PLA2G1C increased EV71 virus replication 2-3-fold, while there was a ~2-fold increase when STRADA and ZNF205 were KD (Fig 2B). Little is known about how these host genes and how they may affect EV71 replication. It is possible that KD of more than one gene may have synergistic or additive effects; however this is not well-understood and was not tested in this study. The Cyclin N-Terminal Domain Containing 2 (CNTD2) gene is orphan cyclin where it has a predominantly nuclear location and it is implicated in transcription regulation as described for other cyclins [26]. It is known that the EP300 gene behaves as a histone acetyltransferase and regulates transcription [27]. EMX2 is a gene that encodes a homeobox-containing transcription factor and possibly regulates mRNA transport or translation [28]. The glucagon receptor gene (GCGR) has a role in glucose homeostasis and is implicated to modulate the activity of adenylate cyclase [29, 30]. The N-acetyltransferase 9 (NAT9) gene is involved in the catalysis of the transfer of an acetyl group to a nitrogen atom on the acceptor molecule [31]. NDUFA9 is a subunit of the hydrophobic protein fraction of the NADH: ubiquinone oxidoreductase, the first enzyme complex in the electron transport chain located in the inner mitochondrial membrane [32, 33]. Phospholipase A2, group 1B (PLA2G1B) is an enzyme that catalyzes the release of fatty acids from glycero-3-phosphocholines [34]. Pyrroline-5-carboxylate reductase (PYCR1) is a mitochondrial enzyme that catalyzes proline biosynthesis reducing pyrroline-5-carboxylate to L-proline [35]. Protein transport protein Sec61 subunit gamma (SEC61G) may function to aid protein translocation in the endoplasmic reticulum [36]. The SVOPL gene is poorly annotated and thought to function as a substrate-specific transmembrane transporter [37]. There is experimental evidence at the protein level that zinc finger protein (ZNF)-205 may be involved in transcriptional regulation [38]. For HEp-2 cells (Fig 2B), KD of CNTD2, EMX2, EP300 and PLA2G1B had a 2-3-fold increases in EV71 replication.

HAV viruses are in the picornavirus family [39]. HAV replicated to substantially higher levels in Vero cells (Fig 2C), and to elevated levels in HEp-2 cells (Fig 2D). KD of CNTD2, COQ9, GCGR, NEU2, and PYCR1 genes in Vero cells led to ~100 - >200-fold increases in replication. Similarly, NDUFA9, NEU2, SEC61G, and ZNF205 were found to be important for replication in HEp-2 cells (Fig 2D). KD of NDUFA9, NEU2, SEC61G, and ZNF205 led to 50-100-fold increase in HAV replication. Of note, the NADH dehydrogenase 1 alpha subcomplex subunit 9 (NDUFA9) gene is thought to be an accessory subunit of the mitochondrial membrane respiratory chain NADH dehydrogenase [32]. The NEU2 gene catalyzes the removal of sialic acid from glycoproteins and oligosaccharides [40]. It is not clear how these host genes affect HAV replication. EP300 functions as histone acetyltransferase and regulates transcription via chromatin remodeling [41].

**Fig 3:**
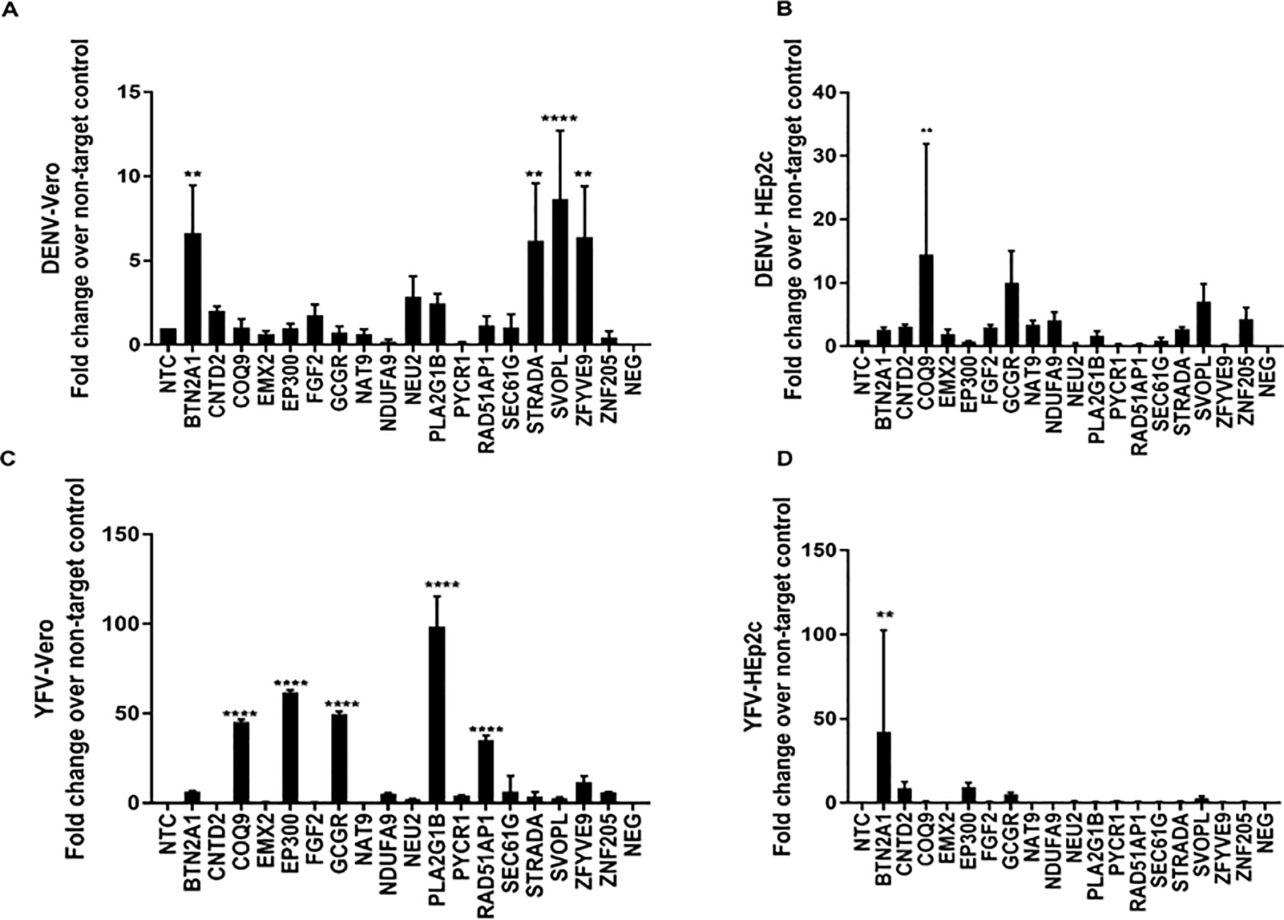
siRNA identifies cell type specificity of genes that modulate replication of Dengue virus (DENV) and Yellow fever virus (YFV). Vero / HEp2-c cells were reverse transfected with non-targeting control (NTC) / gene specific on-target plus (OTP) siRNAs (50nM) for 48h followed by infection with DENV (A and B) or YFV (C and D). Effect of gene knockdown on viral replication is shown as fold-change relative to NTC from pooled samples. Error bars represent ±SEM from two independent experiments. * p<0.05 **p<0.01 ***p<0.001 ****p<0.0001.

Dengue virus (DENV), a member of the *Flaviviridae* family, is transmitted by *Aedes* mosquitos [42]. Based on antibody neutralization, four serotypes (DENV-1, DENV-2, DENV-3, and DENV-4) are evident [43]. KD of BTN2A1, STRADA, SVOPL and ZFYVE9 increased DENV-1 replication in Vero cells (Fig 3A), and KD of COQ9, GCGR, and SVOPL substantially increased virus replication in HEp-2 cells (Fig 3B). These findings indicate that Vero cells and Hep-2 cells are permissive to DENV-1 with 2-3-fold higher virus replication following KD of key genes in HEp-2 cells.

Yellow fever virus (YFV) is also transmitted to humans by Aedes mosquitos [44]. As expected, IFN-deficient Vero cells were more susceptible compared to HEp-2 cells. KD of COQ9, EP300, GCGR, PLA2G1B or RAD51AP1 resulted in enhanced YFV replication (~50-100-fold) in Vero cells (Fig 3C). KD of PLA2G1B had the greatest effect on virus replication, ~100-fold over NTC. YFV replication in HEp-2 cells was diminished relative to Vero cells, with peak titers following KD of BTN2A1 ~40-fold (Fig 3D). As noted, most gene functions as they relate to virus replication remain unknown.

**Fig 4:**
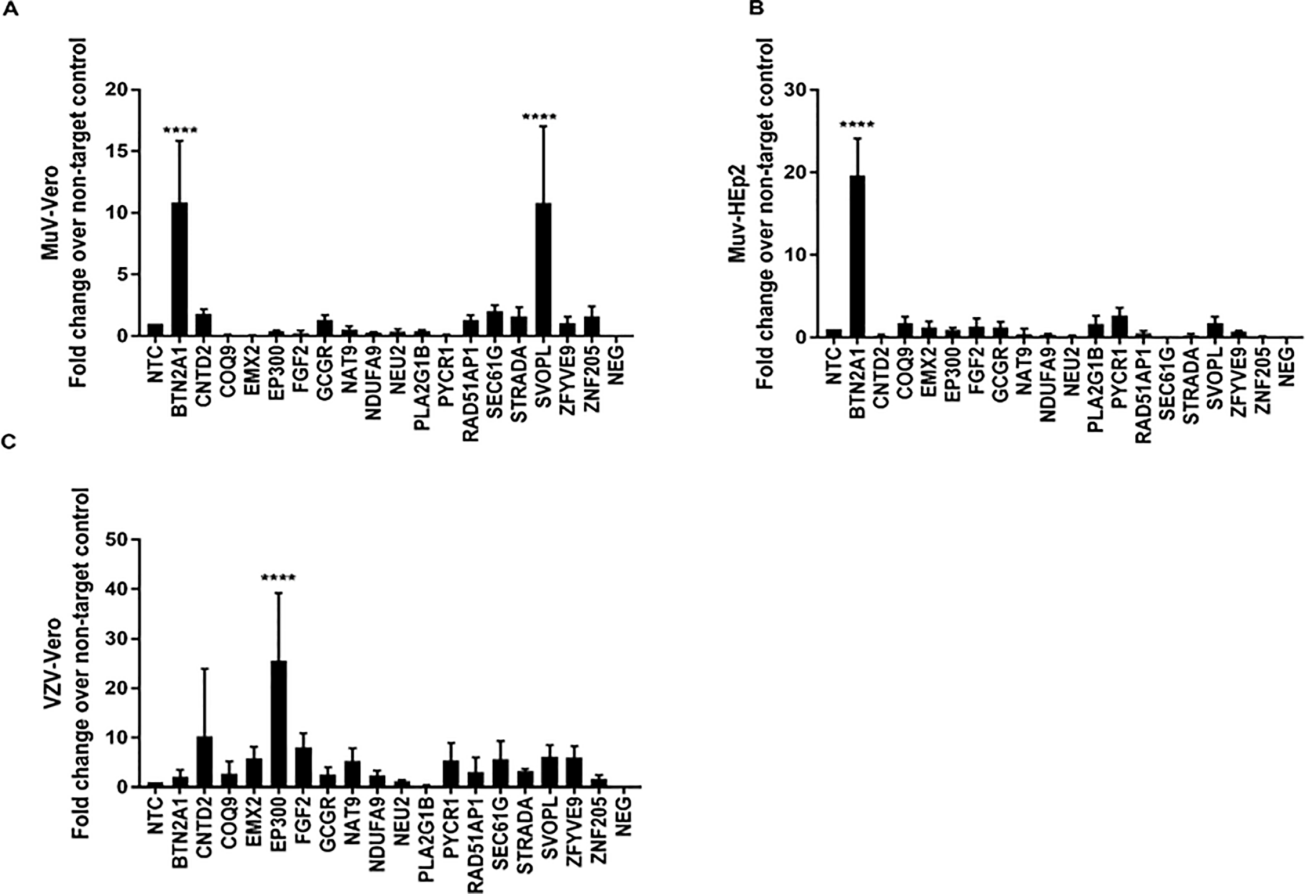
siRNA identifies cell type specificity of genes that modulate replication of mumps virus (MuV) and varicella zoster virus (VZV). Vero / HEp2-c cells were reverse transfected with non-targeting control (NTC) / gene specific on-target plus (OTP) siRNAs (50nM) for 48h followed by infection with MuV (A and B) or VZV (C) (MOI = 0.1). Effect of gene knockdown on viral replication is shown as fold-change relative to NTC from pooled samples. Error bars represent ±SEM from two independent experiments. * p<0.05 **p<0.01 ***p<0.001 ****p<0.0001.

Mumps virus (MuV) is typically propagated in Hep-2 cells [45] as it does not grow well in Vero cells [46]. We examined the permissiveness of MUV for Vero cells and HEp-2 cells having KD of select host genes (Table 3). KD of BTN2A1 and SVOPL dramatically increased MUV propagation in Vero cells (Fig 4A), whereas only KD of BTN2A1 was associated with enhanced virus replication in HEp-2 cells (Fig 4B). KD of BTN2A1 and SVOPL led to increased MUV replication ~10-fold in Vero cells. BTN2A1 KD in HEp-2 cells enhanced MUV replication ~20-fold (Fig 4B).

Varicella zoster (VZV) is the causative agent of chickenpox/shingles and is a DNA virus and member of α-herpesvirus family [47, 48]. VZV is a component of several vaccines including the combined measles, mumps, rubella, and varicella (MMRV) vaccine [47], and newer vaccines such as live-attenuated herpes zoster vaccine for adults >50 years [49]. Vero cells were superior to HEp-2 cells as Vero cells could propagate VZV, thus they were chosen for analysis. KD of CNTD2 and EP300 resulted in a 10-30-fold increase in VZV replication.

**Fig 5:**
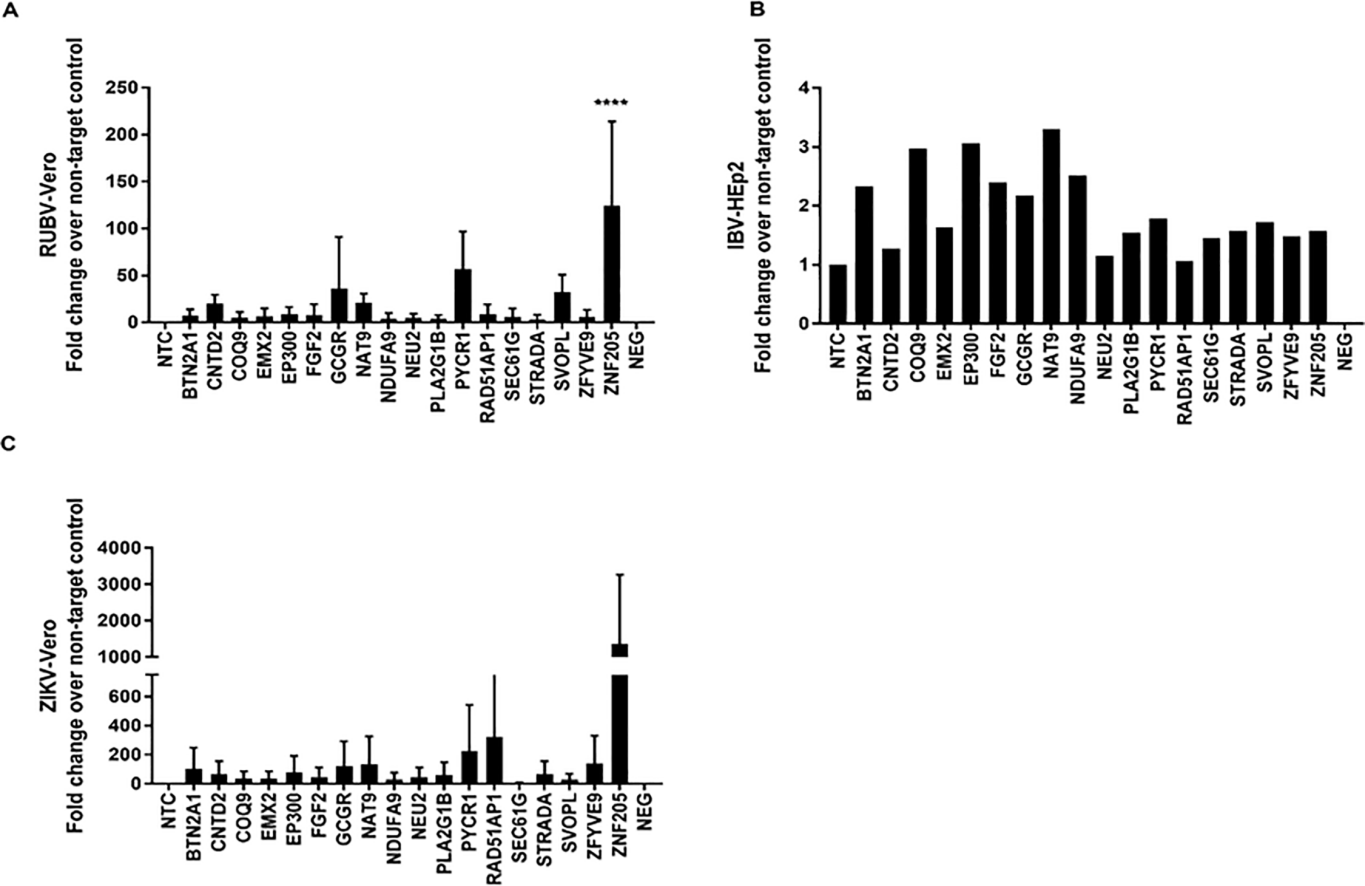
siRNA identifies genes that modulate replication of rubella virus (RUBV) and influenza B virus (IBV) and Zika virus (ZIKV). Vero cells were reverse transfected with non-targeting control (NTC) / gene specific on-target plus (OTP) siRNAs (50nM) for 48h followed by infection with RUBV (A) or IBV (B) or ZIKV (strain PVRABC) (C). Effect of gene knockdown on viral replication is shown as fold-change relative to NTC from pooled samples. Error bars represent ±SEM from two independent experiments. * p<0.05 **p<0.01 ***p<0.001 ****p<0.0001.

Rubella virus (RUBV) is in the family Togaviridae [50]. RUBV is an important human pathogen associated with it birth defects, known congenital rubella syndrome (CRS), but through use of vaccines rubella and CRS are controlled [50]. We investigated if RUBV would grow in Vero cell and HEp-2 cells, but found it could only be propagated in Vero cells (Fig 5A). This is likely related to features of the antiviral IFN pathway [51] but was not specifically investigated. KD of GCGR, PYCR1, SVOPL, or ZNF205 led to 10->100-fold increases in RUBV, where KD of ZNF205 led to highest increases in RUBV.

There are four types of influenza viruses, A, B, C and D, where human influenza A and B viruses cause seasonal epidemics and influenza type C infections generally cause a mild respiratory illness, and influenza type D viruses primarily affect cattle and are not known to infect or cause illness in people [52]. Currently, two lineages of type B influenza virus (IBV) co-circulate in the world, thus the key host genes that affect IBV replication were determined in Vero cells which are an FDA-approved vaccine cell line used to propagate flu viruses (Fig 5B). KD of BTN2A1, COQ9, EP300, FGF2, GCGR, NAT9, and NDUFA9 genes had a ~2-3-fold increased IBV replication. Fig 5B shows the results from one independent plaque assay where n = 3 wells/gene KD were evaluated, and the fold-change over the NTC is shown. Lastly, Zika virus (ZIKV) which belongs to the Flaviviridae family and is spread by mosquitoes [53], was evaluated for replication in Vero cells (Fig 5C) as ZIKV did not replicate in HEP-2 cells. Two ZIKV strains (PVRABC59 and lbH30656) were examined. These ZIKV strains only grew in Vero cells which is consistent with their IFN-sensitivity in HEp-2 cells [54]. In Vero cells, KD of PYCR1, RAD51AP1, and ZNF205 lead to a 200-1000-fold increase in ZIKV virus titers, with the highest increases for KD of ZNF-205. The role for these genes restricting virus ZIKV replication is not known. However, it has been shown that loss of PYCR1 function leads to decreased mitochondrial membrane potential and increased adequate susceptibility to apoptosis under oxidative stress [55]. These finding show that there are a spectrum host genes that restrict cell tropism and virus replication. All raw data used to summarize data shown in Figs 1–5 is available as supplementary data (S1 Fig).

## Discussion

To make a universal cell line that is receptive to a range of viruses, one should identify host genes that influence intrinsic immunity and help determine restriction or permissiveness toward virus infection and replication. There is convincing evidence that many viruses have evolved not only to avoid the apoptosis-mediated viral elimination, but also to exploit and even activate it to facilitate their lifecycle. As one example, influenza proteins NS1 and PB1-F2 act as apoptosis promoters [56, 57], and it was found that caspase-3 activation was needed for efficient influenza virus replication [58–61]. Each virus has found its way to survive, and co-opt the host cell for replication.

Vero cells are one of the first continuous cell lines that were used for vaccine production which was helped by their lack of anti-viral IFN signaling [8, 11]. This feature makes the Vero cell line more permissive for a wide spectrum of viruses [6, 62]. In this study, we KD the top genes discovered from screening and validating a siRNA genome-wide screen of IAV, RV, or PV (Tables 1–2) with the goal of finding the top host genes in Vero cells and HEp-2 cells (Table 3) that could be used to create a universal vaccine cell line. We have shown that Vero cells transfected with siRNAs to KD BTN2A1, CNTD2, EP300, GCGR, PYCR1 and ZNF205 will support enhanced virus replication of most viruses examined here including PV, RV, IAV, DENV, HAV, EV71, MUV, RUBV, YFV, VZV, or ZIKV.

Stand out findings from the results are that 1) it may be possible to create an enhanced universal cell line permissive for many viruses, 2) the Vero cell lines are generally better at propagating viruses than HEp-2 cells, 3) there is increased virus replication is in the KD cell lines, and 4) virus strain differences affect the outcome and magnitude of virus replication. For example, this was evident between ZIKV strains PVRABC59 and lbH30656 which had nearly 5 x10^7^ PFU/mL and 2 x 10^6^ PFU/mL between strains, respectively, using the same propagation conditions. The reasons for these differences are not presently known.

In this study, we evaluated viruses with known strain variation, and expected these differences to affect the host genes linked to virus replication as was discovered for PV studies [1]. Another example of antigenically distinct viruses are DENV which has been classified into four individual serotypes (DENV 1–4) [63, 64]. DENV like many other viruses has evolved multiple mechanisms to evade the innate immune response and increase virus replication. It is clear that universal vaccination can have major impacts on diseases such as PV, MUV, and RUBV [65, 66]. One the other hand, vaccine-preventable diseases such as IAV continue to cause significant disease because of use of our inadequate use of vaccines [67]. Advances in vaccine technology are now providing the opportunity to target new diseases and create enhanced vaccine cell lines. Making a cell line that allows for reduced costs is a rate-limiting feature that will drive prioritization and going forward. For example, RV is a common cause of severe gastroenteritis in children. RV causes a large disease burden ($US 2 billion in 2007) [4], where morbidity and mortality occurs in malnourished infants in low and middle income countries. Vaccines typically confer 85–100% protection against severe disease, while in low income regions protection is around 46–77%, thus the RV vaccination program must rely upon sustained vaccine efficacy level against evolving strains. Cost effectiveness depends largely on the RV mortality rate and the price of the vaccine in relation to the per capita gross domestic product. An enhanced vaccine cell line like Vero cells could save millions of dollars and improve the quality life worldwide.

## Materials and methods

### Cells and viruses

A siRNA screen was performed using Vero cells (African green monkey kidney cells; received from the Centers of Disease Control and Prevention (CDC, Atlanta) who obtained them from the Serum Institute of India (SI) [68] and HEp-2 cells (human laryngeal carcinoma, HeLa-derivative; received from the Centers of Disease Control and Prevention, Atlanta [69]. Vero cells were cultured at 37°C and 5% CO2 in Dulbecco’s modified Eagle’s medium (DMEM; HyClone, GE Healthcare) supplemented with 10% fetal bovine serum (FBS; HyClone). The same passage number of Vero cells and HEp-2 cells was used in validation studies to minimize differences and aid interpretation. A master cell bank was created to ensure that all experiments were performed using a consistent low number of cell passages. PV (Sabin-2) was obtained from National Institute for Biological Standards and Control, Health Protection Agency, Potters Bar, United Kingdom. RV strain RV3 was obtained from the CDC and propagated in MA104 cells (rhesus monkey epithelial cell line; clone 1, ATCC CRL-2378.1 [70]. Peak ELISA antigen levels was determined as previously described [3]. Briefly, Vero cells were infected with RV (MOI = 0.2), the cells fixed prior to performing an immunofluorescent ELISA. For immunofluorescent staining, fixed plates were stained using a primary polyclonal rabbit anti-RV antibody (Rab A-SA11, Australia) the plates washed, blocked, and were then a goat anti-rabbit Alexa 488 antibody used for detection with a Beckman Coulter Paradigm spectrophotometer. Additionally, the percent of infected cells per well was determined relative to that of the non-targeting control by additional staining of the plate with DAPI to detect cell nuclei and scanning on a Cellomics Array scanner. IAV (A/WSN 33) and influenza B/Malaysia/2506/2004 were propagated in embryonated chicken eggs. DENV-1 (Hawaii, BEI resources NR-82) was grown in Vero cells [71]. The YFV 17 D vaccine strain (BEI resources NR-116) was grown in Vero cells [71]. HAV (ATCC- VR-2266) was grown in MRC-145 cells [72]. Coxsackie Virus B5 (EV71; ATCC VR-185) was grown in Vero cells [73]. MUV virus (ATCC VR-1438) was grown in HEp-2 cells [74]. RUBV (ATCC VR-1359) was grown in Vero cells [75]. VZV (human herpesvirus 3 ATCC VR-1367) was grown in MRC-145 cells [76]. ZIKV (strains PVRABC59 and lbH30656 were tested) in Vero cells [77].

### Cellular growth, viability and cytotoxicity

Vero and HEp-2 cells were screened for cellular viability and cytotoxicity following siRNA transfection by CellTiter96 Non-Radioactive Cell Proliferation Assay Kit and ToxiLight bioassay (Lonza, Walkersville, MD) following manufacturer’s instructions. Cellular toxicity was evaluated at 0 and 48 h post-transfection. Data was normalized to siTOX control (100% cytotoxicity). siRNAs which resulted in >10% toxicity were excluded from the screen. Cell growth assessment revealed no significant differences in cell adherence or doubling time between KD and wild type cells.

### Human siRNAs

ON-TARGETplus siRNAs (GE Healthcare, Lafayette, CO) combine sense-strand inactivation with a novel seed-region modification and were used to target the host genes of interest (Table 1). Non-targeting controls allowed assessment of non-specific effects. Pooled siRNA controls were used for reduction of off-targeting with Vero cells, and for use with SMARTpool siRNA reagents that were used for validation.

siRNAs targeting the 18 host genes of interest (Table 1) were reverse transfected in triplicate into HEp-2 cells or Vero cells at a final concentration of 50 nM using 0.3% DharmaFECT-4, and triplicate experiments were done three independent times unless noted otherwise. Briefly, On-TARGETplus siRNAs were mixed with DharmaFECT 4 reagent in serum-free medium (Opti-MEM; Invitrogen Inc., Carlsbad, CA) and incubated at room temperature for 20 min. A NTC siRNA (GE Healthcare) and a PV, RV or IAV type-specific positive siRNA control target were included [78]. Vero cells were subsequently added (9,000 cells/well) with DMEM supplemented with 10% FBS. Transfected cells were cultured at 37°C and 5% CO2. After 48h, the medium was removed and cells were infected with PV (MOI = 0.1) in DMEM supplemented with 2% FCS and 1% penicillin-streptomycin. In some cases, 24h pi; the plates were frozen at 80°C and stored until analysis of antigen content by ELISA. For siRNAs targeting the other viruses (Table 1), similar methods were used [1, 18]. The validated gene hits from IAV, PV and RV studies were analyzed for the top 6 PV, IAV virus, or RV hits, and several end-point assays [19] such as plaque assay were re-confirmed. For PV, RV and IAV, all assays used an endpoint of day 3 pi with a MOI = 0.1. ZIKV was at day 5 pi at MOI = 0.1. For all other others examined an endpoint of day 7–10 pi was used. In all siRNA studies, there was >95 gene KD (Table 2) which is consistent with the manufacturer assertions (Dharmacon GE).

### RT-qPCR

qPCR was performed to evaluate that KD took place with each of the siRNAs [79]. We examined KD of the genes (Table 1) in HEp-2 and Vero cells using the comparative CT method (ΔΔCT) for relative quantitation for measuring siRNA-induced KD of a particular gene using TaqMan, and >95% gene KD was confirmed (Table 2). The RNA from the siRNA-transfected cells was assessed by measuring the RNA levels of the GAPDH gene, according to an endogenous 18S RNA. A NTC siRNA-transfected was also assessed to the experimental samples to assess the negative control samples. The ΔΔCT was used to calculate the percent remaining gene expression and the percent knockdown. Since the Vero genome is not fully annotated, we designed Vero specific primers using available contigs, amplified the regions from normal Vero cDNA using high fidelity Q5 Polymerase (New England Biolabs) and cloned the inserts into TOPO TA vectors which were then sequence validated. Amplifications conditions were optimized as needed.

### Statistics

The data are expressed as the fold-change over the non-targeting control, except for comparison of ZIKV strains which are mean of n = 3 experiments ±SE. Statistical analyses were performed using GraphPad Prism software using one-way ANOVA (reporter studies) with post hoc Dunnett’s test. Error bars represent ±SEM of three independent experiments.

### Ethics statement

The University of Georgia (UGA) Institutional Biosafety Committee approved all research involving embryonated chicken eggs. The embryonated eggs were obtained from the Southeast Poultry Research center at UGA. For propagation of influenza viruses, specific pathogen-free eggs were used 7–8 days after fertilization.

## Supporting information

S1 Fig Raw viral titers/ absorbance values for all figures in the manuscript. Supplementary excel file contains all raw data from viral titer assays in this manuscript. All assays were done in multiple technical replicates with either two or three biological replicates. Each worksheet summarizes fold change in viral titers relative to NTC for Vero and /or HEp-2 cells. Worksheet labels indicate virus-cell line-Figure panel.
(XLSX)
